# Development and Analysis of a Quantitative Mathematical Model of Bistability in the Cross Repression System Between APT and SLBO Within the JAK/STAT Signaling Pathway

**DOI:** 10.3389/fphys.2020.00803

**Published:** 2020-07-28

**Authors:** Alyssa Berez, Bradford E. Peercy, Michelle Starz-Gaiano

**Affiliations:** ^1^Department of Mathematics and Statistics, University of Maryland Baltimore County, Baltimore, MD, United States; ^2^Department of Biological Sciences, University of Maryland Baltimore County, Baltimore, MD, United States

**Keywords:** JAK/STAT, *Drosophila melanogaster*, border cell migration, mathematical model, bistability

## Abstract

Cell migration is a key component in development, homeostasis, immune function, and pathology. It is important to understand the molecular activity that allows some cells to migrate. *Drosophila melanogaster* is a useful model system because its genes are largely conserved with humans and it is straightforward to study biologically. The well-conserved transcriptional regulator Signal Transducer and Activator of Transcription (STAT) promotes cell migration, but its signaling is modulated by downstream targets Apontic (APT) and Slow Border Cells (SLBO). Inhibition of STAT activity by APT and cross-repression of APT and SLBO determines whether an epithelial cell in the *Drosophila* egg chamber becomes motile or remains stationary. Through mathematical modeling and analysis, we examine how the interaction of STAT, APT, and SLBO creates bistability in the Janus Kinase (JAK)/STAT signaling pathway. In this paper, we update and analyze earlier models to represent mechanistically the processes of the JAK/STAT pathway. We utilize parameter, bifurcation, and phase portrait analyses, and make reductions to the system to produce a minimal three-variable quantitative model. We analyze the manifold between migratory and stationary steady states in this minimal model and show that when the initial conditions of our model are near this manifold, cell migration can be delayed.

## 1. Introduction

The acquisition of cellular migration plays a critical role in both normal and pathological development. A better understanding of the processes cells undergo as they transition from a stationary state to a migratory state is thus of broad interest. Studying the mechanics of how cohorts of cells move together introduces additional complexities, and existing models of collectively migrating cells differ greatly (Peercy and Starz-Gaiano, 2020; Stuelten et al., 2018; Aman and Piotrowski, 2010; Saadin and Starz-Gaiano, 2016; Olson and Nechiporuk, 2018; Leonard and Taneyhill, 2020; Friedl and Mayor, 2017). To study cell migration, some scientists turn to an experimental model system amenable to both genetic analysis and live imaging. In *Drosophila melanogaster* during oogenesis, a set of cells called border cells develop within a layer of follicle cells and later become migratory, leaving the nearby epithelial cells behind as they move to the oocyte (Montell et al., 2012; Saadin and Starz-Gaiano, 2016). Experimentalists have discovered much of the molecular regulation that governs this process and what causes some border cells to become motile while others remain stationary, including the primary biochemical and molecular pathways. We are interested in advancing a mathematical model of these pathways, which could have implications on acquisition of cell motility in animals in general.

**Figure 1:**
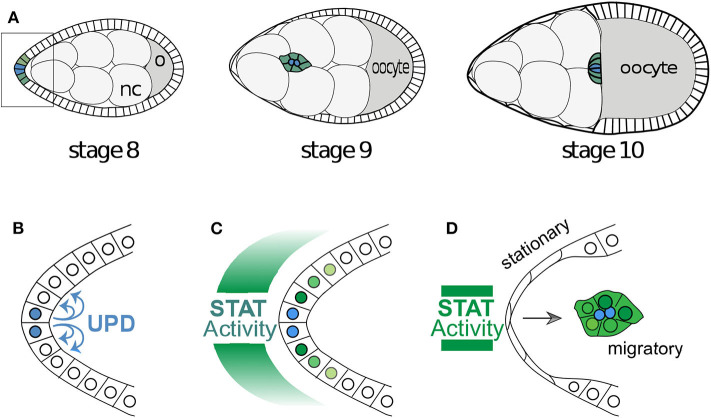
Egg development and the migration of border cells. **(A)** A cartoon of the development of a *Drosophila* egg chamber and the movement of the border cells between the nurse cells (nc). The outer edge of the egg chamber is made up of epithelial follicle cells and the box outlines the cells in **(B)**. **(B)** The signaling molecule Unpaired is secreted from the polar cells and **(C)** induces a gradient of STAT activity across the anterior epithelium. Often the follicle cells very close to the polar cells assume the identity of border cells and **(D)** become motile and migrate as a cluster toward the oocyte. The cells with below-threshold levels of STAT activity shut the signaling off in a switch like manner.

**Figure 2:**
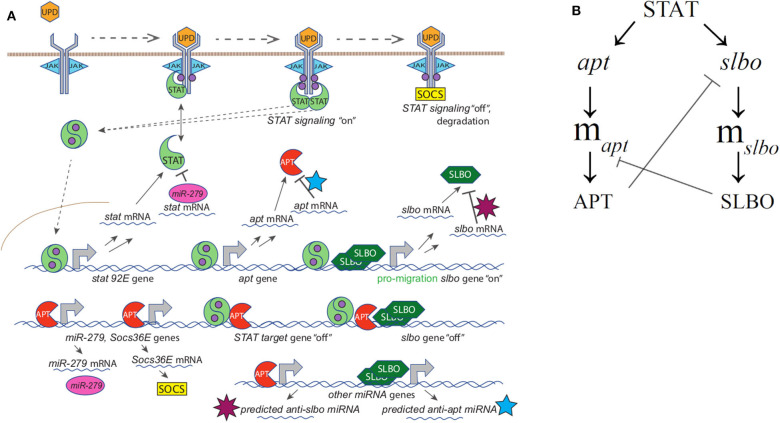
**(A)** Full mechanistic diagram of JAK/STAT pathway **(B)** Cross-repression system of APT and SLBO. STAT activates *apt* and *slbo* transcription leading to the production of *apt* and *slbo* mRNA and translation into APT and SLBO proteins. SLBO represses *apt* translation while APT suppresses *slbo* transcription and function. High APT levels make the cell stationary and high SLBO levels make the cell motile. However, which state dominates cannot be determined from this qualitative diagram alone.

The Janus Kinase/Signal Transducer and Activator of Transcription (JAK/STAT) signaling pathway has been shown by previous studies to be crucial in the motility of border cells, as well as in stem cells and immune response (Montell et al., 2012; Arbouzova and Zeidler, 2006; Trivedi and Starz-Gaiano, 2018; Amoyel and Bach, 2012; Amoyel et al., 2014). The JAK/STAT pathway is well-conserved from fruit flies to humans. Anterior polar cells in the *Drosophila* egg chamber (see Figures 1, 2A) secrete the cytokine Unpaired (UPD), which acts as the ligand for the transmembrane Domeless receptor in neighboring follicle cells. UPD is predicted to form a gradient across the adjacent cells (Van De Bor et al., 2011; Xi et al., 2003; Starz-Gaiano et al., 2008). The binding of UPD to Domeless activates JAK, leading to the phosphorylation of the activated JAK/receptor complex. The activated complex then recruits and phosphorylates STAT. The phosphorylated STAT dimerizes, moves to the nucleus, and acts as a transcription factor for specific target genes.

A key target of STAT is the C/EBP transcription factor Slow Border Cells (Slbo) (Montell et al., 1992; Starz-Gaiano et al., 2008). Cells specified by high levels of SLBO to become the border cells respond to chemoattractants that activate two receptor tyrosine kinases (RTKs), which turn on signaling cascades that promote directional movement (Montell et al., 2012; Stuelten et al., 2018; Saadin and Starz-Gaiano, 2016; Friedl and Mayor, 2017). Downstream of the RTKs, guided motility is governed in border cells largely through regulation of the actinomyosin cytoskeleton via the Rho GTPase, Rac, and myosin phosphorylation states, with coordinated changes in cell-cell adhesion mediated by E-cadherin (Stuelten et al., 2018; Saadin and Starz-Gaiano, 2016; Montell et al., 2012; Chen et al., 2020). The follicle cells receiving sufficiently high levels of UPD—not necessarily just those in closer proximity to the polar cells depending on the extracellular geometry (Manning et al., 2015)—turn on a higher level of STAT activity and become motile border cells while nearby cells with lower levels do not. Interestingly, high STAT activity in ovarian follicle cell is sufficient to induce motility in usually stationary lateral follicle cells (Silver and Montell, 2001).

We focus on the protein products from two genes activated by STAT: Apontic (APT) and SLBO. SLBO promotes migratory behavior and an insufficient amount of SLBO prevents motility (Montell et al., 1992). APT protein is a transcription factor that downregulates the function of JAK/STAT and SLBO, and thus inhibits migration (Starz-Gaiano et al., 2008). APT acts as a feedback inhibitor on the JAK/STAT pathway, and this process is mediated by APT's activation of a microRNA that reduces STAT protein and activity (Yoon et al., 2011). APT also activates expression of *Socs36E*, which downregulates STAT signaling via a degradation pathway (Monahan and Starz-Gaiano, 2015). APT and SLBO exhibit cross-repressional behavior (Starz-Gaiano et al., 2008, 2009). APT directly represses *slbo* transcription while SLBO only decreases the level of expression of APT protein. The dominating protein in a given cell determines the cell fate: stationary or motile. This creates what appears to be a bistable system, as cells that receive intermediate amounts of STAT have the potential to reach either fate (Starz-Gaiano et al., 2009; Rorth, 1994). While it is reasonable based on the non-linearities in the system to model this cell fate regulation as bistability, *in vivo* experiments to support this are challenging and we are not aware of any particular experiments that have been done to confirm bistability. By identifying the conditions under which bistability occurs, modeling can help to design experimental protocols to confirm bistability.

We base our model on the mechanistic model built by Ge et al. (2012). Focusing on the cross-repression system of APT and SLBO, they built a mathematical model using elementary interactions to identify which components of the system are sufficient for bistability. Depending on the strength of the UPD signal and thus the level of STAT activity, each border cell can become motile (SLBO dominates) or remain stationary (APT dominates). Cells with an intermediate level of STAT activity may fall above or below the threshold necessary for mobility.

Ge and Stonko created a 15-variable model including many mechanisms of STAT regulation (see Figure 2A) as well as the cross-repression system of APT and SLBO (see Figure 2B) with sufficient elements, specifically cooperativity in SLBO repressing *apt* mRNA translation, to cause bistability. We do not know the mechanism for this, but we suspect SLBO activates expression of a microRNA that mediates the effect. Ge et al. (2012) identified several miRNAs in the *Drosophila* genome that have upstream binding sites for SLBO activation and seed sequences that would target the apt mRNA 3′ untranslated regions.

To describe briefly the full model, in Equations (1) and (2) JAK (J) is altered to an enzymatically active state, J^*^, in the presence of UPD (U). Further in Equation (1) J^*^ enzymatically activates two STAT monomers (S) to create the activated STAT dimer ($S_{2}^{*}$) shown for STAT variables and complexes in Equations (3)–(6) with *c*_1_ denoting the complex between activated JAK (J*) and STAT (S). Equation (5) represents APT sequestering $S_{2}^{*}$ in *c*_2_. In Equations (7) and (8) APT (A) and SLBO (B) are produced from their mRNA and degraded, while A binding with $S_{2}^{*}$ is accounted for. The mRNA production of *stat* (*m*_σ_), *apt* (*m*_α_), and *slbo* (*m*_β_), is shown in Equations (7)–(9) based on a constant basal production and transcribing or probabilistic fraction based on non-transcribing *stat* (σ), *apt* (α), and *slbo* (β) respectively, along with degradation. In Equation (9) B cooperatively enhances *m*_α_ degradation, while in Equations (10) and (11) A enhances *m*_σ_ and *m*_β_ degradation. The gene state dynamics are shown in Equations (12)–(15), with $S_{2}^{*}$ inducing transcription of σ, α, and β, while binding with A puts *slbo* into a repressed state (β^*R*^). Model variables for Equations (1)–(15) from Ge et al. (2012) are in Table 1.

**Table 1:** 15-variable model variables.

**Variable**	**Description and Units**
J^*^	Activated JAK protein (nM)
*J*	JAK protein (nM)
*S*	STAT protein monomer (nM)
*c*_1_	J^*^ and *S* complex (nM)
*c*_2_	$S_{2}^{*}$ and *A* complex (nM)
$S_{2}^{*}$	Activated STAT protein dimer (nM)
*A*	APT protein (nM)
*B*	SLBO protein (nM)
*m*_α_	APT mRNA (nM)
*m*_β_	SLBO mRNA (nM)
*m*_σ_	STAT mRNA (nM)
α	Proportion of inactive apt genes
β	Proportion of inactive slbo genes
β^*R*^	Proportion of repressed slbo genes
σ	Proportion of inactive stat genes

The 15-variable model is:

(1) $$\begin{array}{r} \frac{dJ^{*}}{dt}=k_{UJ}^{f}UJ-k_{UJ}^{b}J^{*}-k_{c_{1}}^{f}J^{*}S^{2}+k_{c_{1}}^{b}c_{1}+k_{c_{1}}c_{1} \end{array}$$

(2) $$\begin{array}{r} \frac{dJ}{dt}=-k_{UJ}^{f}UJ+k_{UJ}^{b}J^{*} \end{array}$$

(3) $$\begin{array}{r} \frac{dS}{dt}=-2k_{c_{1}}^{f}J^{*}S^{2}+2k_{c_{1}}^{b}c_{1}+2k_{S_{2}^{*}}S_{2}^{*}+k_{s}m_{\sigma }-\delta_{S}S \end{array}$$

(4) $$\begin{array}{r} \frac{dc_{1}}{dt}=k_{c_{1}}^{f}J^{*}S^{2}-k_{c_{1}}^{b}c_{1}-k_{c_{1}}c_{1} \end{array}$$

(5) $$\begin{array}{r} \frac{dc_{2}}{dt}=k_{S_{2}^{*}A}^{f}S_{2}^{*}A-k_{S_{2}^{*}A}^{b}c_{2} \end{array}$$

(6) $$\begin{array}{r} \frac{dS_{2}^{*}}{dt}=k_{c_{1}}c_{1}-k_{S_{2}^{*}}S_{2}^{*}-k_{S_{2}^{*}A}^{f}S_{2}^{*}A+k_{S_{2}^{*}A}^{b}c_{2} \end{array}$$

(7) $$\begin{array}{r} \frac{dA}{dt}=k_{A}m_{\alpha }-\delta_{A}A-k_{S_{2}^{*}A}^{f}S_{2}^{*}A+k_{S_{2}^{*}A}^{b}c_{2} \end{array}$$

(8) $$\begin{array}{r} \frac{dB}{dt}=k_{B}m_{\beta }-\delta_{B}B \end{array}$$

(9) $$\begin{array}{r} \frac{dm_{\alpha }}{dt}=k_{m_{\alpha }}\left(1-\alpha \right)-\delta_{m_{\alpha }}m_{\alpha }+m_{\alpha }^{o}-\delta_{B_{\alpha }}B^{2}m_{\alpha } \end{array}$$

(10) $$\begin{array}{r} \frac{dm_{\beta }}{dt}=k_{m_{\beta }}\left(1-\beta -\beta^{R}\right)-\delta_{m_{\beta }}m_{\beta }+m_{\beta }^{o}-\delta_{A_{\beta }}Am_{\beta } \end{array}$$

(11) $$\begin{array}{r} \frac{dm_{\sigma }}{dt}=k_{m_{\sigma }}\left(1-\sigma \right)-\delta_{m_{\sigma }}m_{\sigma }+m_{\sigma }^{o}-\delta_{A_{\sigma }}Am_{\sigma } \end{array}$$

(12) $$\begin{array}{r} \frac{d\alpha }{dt}=-k_{\alpha }^{f}S_{2}^{*}\alpha +k_{\alpha }^{b}\left(1-\alpha \right) \end{array}$$

(13) $$\begin{array}{r} \frac{d\beta }{dt}=-k_{\beta }^{f}S_{2}^{*}\beta +k_{\beta }^{b}\left(1-\beta -\beta^{R}\right) \end{array}$$

(14) $$\begin{array}{r} \frac{d\beta^{R}}{dt}=k_{\beta^{R}}^{f}A\beta -k_{\beta^{R}}^{b}\beta^{R} \end{array}$$

(15) $$\begin{array}{r} \frac{d\sigma }{dt}=-k_{\sigma }^{f}S_{2}^{*}\sigma +k_{\sigma }^{b}\left(1-\sigma \right) \end{array}$$

In this paper we analyze and adapt the Ge and Stonko model so that a minimal reduction retains the dynamics of the detailed model. In section 2 we describe the methods we use to establish parameters and for bifurcation analysis. In section 3 we develop the minimal reduced model. In section 4 we analyze bifurcation results, identify the critical stable manifold separating the migratory and stationary steady states, validate experimental results, and compare our model to previous models. We apply this model in the interesting case of controlling microRNA-mediated degradation of *stat* mRNA via APT and show that delays in STAT activation, even to the point of activation failure within a biophysical time span, are possible due to the proximity of the critical UPD level to a limit point bifurcation. We conclude with possible experimentation that could test and improve our model.

## 2. Materials and Methods

In this section, we describe the methods used to analyze our minimal reduced model developed below. We use bifurcation and parameter analysis and identify the stable manifold between the stationary and motile steady states.

### 2.1. Time Course and Bifurcation Analysis

We use XPPAUT (Ermentrout, 2002) to create time course simulations and bifurcation diagrams and to analyze the bistability of the reduced model. We use the stiff integration method to solve our system of ODEs. A bifurcation occurs when a small change in parameter values results in a qualitative change in a system. Bifurcation analysis allows us to identify limit points treating UPD as a parameter, and helps to identify the separatrix between our steady states.

### 2.2. Establishing Parameters

In order to establish a more biophysically realistic model for this study, we researched existing literature to find data to establish parameter values. For some parameters we were able to find data specific to the JAK/STAT pathway or *Drosophila*. For other parameters, related pathways were used to obtain data relevant to this model.

We were able to identify published values for general translation and transcription rates and applied the established rates to the lengths of JAK (encoded by *hopscotch*), STAT (encoded by *Stat92E*), APT, and SLBO genes and proteins (Hargrove et al., 1991; Lewin, 2004). The lengths are found in the fly genome database, Flybase (Thurmond et al., 2019). Protein and mRNA degradation rates have a wide range of average values so JAK, STAT, APT, and SLBO are assumed to conform to this range (Guido et al., 2006; Harris et al., 2011; Nicholson and Nicola, 2013). The protein to DNA binding and dissociation rates of JAK and STAT have been identified in general but not specifically for APT and SLBO (Halford and Marko, 2004; Parsaeian et al., 2013; Yang et al., 2002; Nicholson and Nicola, 2013).

Many signaling pathways, including the JAK/STAT pathway, are regulated by microRNAs (miRNAs) (Lui et al., 2015; Yoon et al., 2011). Ge et al. (2012) identified several microRNAs annotated in the genome (Thurmond et al., 2019; Betel et al., 2007) that have seed sequences corresponding to *apt* and *slbo*, suggesting they are also regulated by this mechanism. This regulation can occur through mRNA degradation, translational inhibition, or other means, making the parameters corresponding to miRNA kinetics difficult to assign. Ge and Stonko addressed this by condensing the various processes by which miRNA can affect mRNA into one degradation rate. We identified a parameter value for this combined effect through information from the model established in Yoon et al. (2011). The rate that *slbo* transitions in and out of its repressed state was also hard to identify due to lack of data, so we utilized the rate given in Ge and Stonko which was adapted from the rate for a different repressor in Harris et al. (2011) and information from Rorth (1994). The base levels of STAT and the total amount of JAK present in border cells were estimated from Yoon et al. (2011); Starz-Gaiano et al. (2008), and McGregor et al. (2002). Lastly, for the rates of STAT independent mRNA production, we again used the original parameter values from Starz-Gaiano et al. (2009). For *slbo*, this rate is most likely negligible. However, *apt* can be activated by means other than STAT. The protein Eyes Absent (EYA) can also activate transcription of *apt* (Starz-Gaiano et al., 2009).

### 2.3. Manifolds Separating Cell Fate Basins of Attraction

One goal in developing the three-variable model was to be able to fully understand the manifold that separates the steady states in the model. For a level of UPD in the bistable region, cells can either become motile or remain stationary depending on the initial conditions of the system.

We visually identified the manifolds by labeling initial conditions according to the steady state to which they converge. This allows us to see the basins of attraction for each steady state. These two stable steady states, one where SLBO dominates and the cell becomes motile and one where APT dominates and the cell remains stationary, are listed in the appendix with values for each variable. In our three-dimensional system we are able to approximate the manifold by fitting a surface to the boundary between the basins of attraction.

A graphical representation of the boundary manifold was achieved by creating a 3-d grid of initial conditions and determining to which steady state each converged within 500 min, a time deemed reasonable from experimental data. Any initial conditions that did not converge by this time were identified as lying near the manifold. We then used the MATLAB curve fitting toolbox to fit a surface to these points, creating an approximation of the manifold between the steady states.

## 3. Developing the Reduced Model

We began by reducing the fixed STAT cross-repression system of APT and SLBO (Equations, 7–10, 12–14) to a two-variable model. In the process of researching biophysically realistic parameters we discovered that the binding rate of STAT to target genes appears to be several orders of magnitude faster than any other process in the system, as seen in Table 2 (Halford and Marko, 2004; Parsaeian et al., 2013; Yang et al., 2002; Nicholson and Nicola, 2013; Karsten et al., 2006; Ekas et al., 2010). For example, $k_{\alpha }^{f}$, $k_{\beta }^{f}$, and $k_{\beta^{R}}^{f}$ are at least three orders of magnitude faster than the translation and transcription kinetics. Additionally, α, β, and β^*R*^ reach equilibrium significantly faster than the other variables. We used time-scale analysis to reduce the system. We made a quasi-steady state approximation for α, β, and β^*R*^ and set those derivatives equal to zero. This allowed us to solve Equations (12), (13), and (14) for α^*^ = 1−α and β^*^ = 1−β−β^*R*^ in terms of APT protein and STAT dimer:

(16) $$\begin{array}{r} \alpha^{*}=\frac{\frac{S_{2}^{*}}{K_{\alpha }}}{\frac{S_{2}^{*}}{K_{\alpha }}+1} \end{array}$$

(17) $$\begin{array}{r} \beta =\frac{1}{\frac{S_{2}^{*}}{K_{\beta }}+1+\frac{A}{K_{\beta^{R}}}} \end{array}$$

(18) $$\begin{array}{r} \beta^{R}=\frac{k_{\beta^{R}}^{f}}{k_{\beta^{R}}^{b}}A\beta =\frac{\frac{A}{K_{\beta^{R}}}}{\frac{S_{2}^{*}}{K_{\beta }}+1+\frac{A}{K_{\beta^{R}}}} \end{array}$$

(19) $$\begin{array}{r} \beta^{*}=1-\beta -\beta^{R}=\frac{\frac{S_{2}^{*}}{K_{\beta }}}{\frac{S_{2}^{*}}{K_{\beta }}+1+\frac{A}{K_{\beta^{R}}}} \end{array}$$

$$\text{with}K_{\alpha }=\frac{k_{\alpha }^{b}}{k_{\alpha }^{f}},K_{\beta }=\frac{k_{\beta }^{b}}{k_{\beta }^{f}},K_{\beta^{R}}=\frac{k_{\beta^{R}}^{b}}{k_{\beta^{R}}^{f}}$$

**Table 2:** Three-variable system parameter values.

**Parameter**	**Symbol**	**Value**	**Units**	**Citation**
Binding rate of UPD to JAK	$k_{UJ}^{f}$	0.0133	min^−1^	Manning et al., 2015; Ghiglione et al., 2002; Wright et al., 2011; Ward et al., 1995; Hilton and Nicola, 1992
Dissociation rate of UPD to JAK	$k_{UJ}^{b}$	0.1	nM· min^−1^	Ghiglione et al., 2002; Wright et al., 2011; Ward et al., 1995; Hilton and Nicola, 1992
Binding rate of J^*^ complex to 2 STAT monomers	$k_{c_{1}}^{f}$	1	min^−1^	Nicholson and Nicola, 2013; Karsten et al., 2006; Ekas et al., 2010
Dissociation rate of J^*^ complex to 2 STAT monomers	$k_{c_{1}}^{b}$	0.1	nM· min^−1^	Nicholson and Nicola, 2013; Karsten et al., 2006; Ekas et al., 2010
Rate of $S_{2}^{*}$ leaving J^*^	*k*_*c*_1__	100	nM· min^−1^	Nicholson and Nicola, 2013; Karsten et al., 2006; Ekas et al., 2010
Dedimerization rate of $S_{2}^{*}$	$k_{S_{2}^{*}}$	0.1	nM^−1^· min^−1^	Nicholson and Nicola, 2013; Karsten et al., 2006; Ekas et al., 2010
Rate of STAT translation	*k*_*S*_	3	min^−1^	Hargrove et al., 1991; Lewin, 2004; Thurmond et al., 2019
Rate of degradation of STAT	δ_*S*_	0.1	min^−1^	Harris et al., 2011; Nicholson and Nicola, 2013
Rate of transcription of *stat*	*k*_*m*_σ__	1	min^−1^	Hargrove et al., 1991; Lewin, 2004; Thurmond et al., 2019
Rate of degradation of *stat* mRNA	δ_*m*_σ__	0.2	min^−1^	Guido et al., 2006; Harris et al., 2011
Base level of *stat* mRNA	$m_{\sigma }^{o}$	0.5	nM· min^−1^	Ge et al., 2012; Silver and Montell, 2001; Ghiglione et al., 2002
Degradation rate of *stat* mRNA due to miRNA induced by APT	δ_*Aσ*_	0.05	nM^−1^· min^−1^	Yoon et al., 2011
Binding rate of $S_{2}^{*}$ to *stat*	$k_{\sigma }^{f}$	1	min^−1^	Halford and Marko, 2004; Parsaeian et al., 2013; Yang et al., 2002; Nicholson and Nicola, 2013; Karsten et al., 2006; Ekas et al., 2010
Dissociation rate of $S_{2}^{*}$ to *stat*	$k_{\sigma }^{b}$	2	nM· min^−1^	Halford and Marko, 2004; Parsaeian et al., 2013; Yang et al., 2002; Nicholson and Nicola, 2013; Karsten et al., 2006; Ekas et al., 2010
Total amount of JAK	*J*_*T*_	0.15	nM	Ge et al., 2012; McGregor et al., 2002
Rate of APT translation	*k*_*A*_	0.298	min^−1^	Hargrove et al., 1991; Lewin, 2004; Thurmond et al., 2019
Rate of transcription of *apt*	*k*_*m*_α__	0.54	nM· min^−1^	Hargrove et al., 1991; Lewin, 2004; Thurmond et al., 2019
Rate of STAT independent production of *apt* mRNA	$m_{\alpha }^{o}$	0.52	nM· min^−1^	Starz-Gaiano et al., 2009
Rate of degradation of APT	δ_*A*_	0.04	min^−1^	Starz-Gaiano et al., 2008; Ge et al., 2012; Harris et al., 2011
Rate of degradation of *apt* mRNA	δ_*m*_α__	0.086	min^−1^	Guido et al., 2006; Harris et al., 2011; Starz-Gaiano et al., 2008
Degradation rate of *slbo* mRNA due to miRNA induced by APT	δ_*A*_β__	0.1	nM^−1^· min^−1^	Ge et al., 2012; this work
Binding rate of $S_{2}^{*}$ to *apt*	$k_{\alpha }^{f}$	100	min^−1^	Halford and Marko, 2004; Parsaeian et al., 2013; Yang et al., 2002; Starz-Gaiano et al., 2008
Dissociation rate of $S_{2}^{*}$ to *apt*	$k_{\alpha }^{b}$	0.66	nM· min^−1^	Halford and Marko, 2004; Parsaeian et al., 2013; Yang et al., 2002
Rate of SLBO translation	*k*_*B*_	0.312	min^−1^	Hargrove et al., 1991; Lewin, 2004; Thurmond et al., 2019
Rate of transcription of *slbo*	*k*_*m*_β__	0.538	nM· min^−1^	Hargrove et al., 1991; Lewin, 2004; Thurmond et al., 2019
Rate of STAT independent production of *slbo* mRNA	$m_{\beta }^{o}$	0.03	nM· min^−1^	Starz-Gaiano et al., 2009
Rate of degradation of SLBO	δ_*B*_	0.04	min^−1^	Harris et al., 2011; Rorth, 1994; Rorth et al., 2000
Rate of degradation of *slbo* mRNA	δ_*m*_β__	0.086	min^−1^	Guido et al., 2006; Harris et al., 2011
Degradation rate of *apt* mRNA due to miRNA induced by SLBO	δ_*B*_α__	0.5	nM^−2^· min^−1^	Ge et al., 2012; this work
Binding rate of $S_{2}^{*}$ to *slbo*	$k_{\beta }^{f}$	100	min^−1^	Halford and Marko, 2004; Parsaeian et al., 2013; Yang et al., 2002; Starz-Gaiano et al., 2008
Dissociation rate of $S_{2}^{*}$ to *slbo*	$k_{\beta }^{b}$	0.66	nM· min^−1^	Halford and Marko, 2004; Parsaeian et al., 2013; Yang et al., 2002; Starz-Gaiano et al., 2008
Rate *slbo* transitions into repressed state	$k_{\beta^{R}}^{f}$	100	min^−1^	Harris et al., 2011; Rorth, 1994
Rate *slbo* transitions out of repressed state	$k_{\beta^{R}}^{b}$	0.522	nM· min^−1^	Harris et al., 2011; Rorth, 1994

Our parameter values indicate that the mRNA processes occur at least twice as fast as the protein processes. This makes a quasi-steady state approximation for *m*_α_ and *m*_β_ plausible. Thus, we have a two-variable model where only APT and SLBO are dynamic.

The two-variable model is:

(20) $$\begin{array}{r} \frac{dA}{dt}=k_{A}\frac{k_{m_{\alpha }}\alpha^{*}+m_{\alpha }^{o}}{\delta_{m_{\alpha }}+\delta_{B_{\alpha }}B^{2}}-\delta_{A}A \end{array}$$

(21) $$\begin{array}{r} \frac{dB}{dt}=k_{B}\frac{k_{m_{\beta }}\beta^{*}+m_{\beta }^{o}}{\delta_{m_{\beta }}+\delta_{A_{\beta }}A}-\delta_{B}B \end{array}$$

(22) $$\begin{array}{r} m_{\alpha }=\frac{k_{m_{\alpha }}\alpha^{*}+m_{\alpha }^{o}}{\delta_{m_{\alpha }}+\delta_{B_{\alpha }}B^{2}} \end{array}$$

(23) $$\begin{array}{r} m_{\beta }=\frac{k_{m_{\beta }}\beta^{*}+m_{\beta }^{o}}{\delta_{m_{\beta }}+\delta_{A_{\beta }}A} \end{array}$$

After establishing the cross-repressional system of APT and SLBO, we reintroduced STAT dynamics to the model. Now we reduce the STAT activation system (Equations 1–6, 11, 15) through a number of assumptions.

First, we ignored the theoretical APT-STAT complex (*c*_2_) as its effects of APT sequestering STAT do not affect the steady state structure of the model, which we proved analytically. Then we used the Michaelis-Menten approximation for the activated JAK (J^*^) conversion of two STAT molecules to an activated STAT dimer ($S_{2}^{*}$). This eliminates the JAK-STAT complex (*c*_1_) and condenses the conversion. We assumed conservation of JAK to eliminate unactivated JAK (J) by defining a constant total JAK as $J_{T}=J+J^{*}$. We also assumed that UPD (U) activation of JAK and STAT activation are fast so J^*^ and $S_{2}^{*}$ can be solved for by quasi-steady state approximations. Lastly, similar to the reduction made for *apt* and *slbo*, the inactive *stat* gene state (σ) and *stat* mRNA (*m*_σ_) were solved by quasi-steady state approximations. These assumptions gave us the following additional approximations:

(24) $$\begin{array}{r} \sigma =\frac{1}{\frac{S_{2}^{*}}{K_{\sigma }}+1}\text{with}K_{\sigma }=\frac{k_{\sigma }^{b}}{k_{\sigma }^{f}} \end{array}$$

(25) $$\begin{array}{r} v_{max}=k_{c_{1}}J^{*} \end{array}$$

(26) $$\begin{array}{r} k_{m}=\sqrt{\frac{k_{c_{1}}^{b}+k_{c_{1}}}{k_{c_{1}}^{f}}} \end{array}$$

(27) $$\begin{array}{r} J^{*}=\frac{k_{UJ}^{f}UJ_{T}}{k_{UJ}^{b}+k_{UJ}^{f}U} \end{array}$$

(28) $$\begin{array}{r} S_{2}^{*}=\frac{1}{k_{S_{2}^{*}}}\frac{v_{max}S^{2}}{S^{2}+k_{m}^{2}} \end{array}$$

Thus, producing our minimal three-variable model in APT, SLBO, and STAT:

(29) $$\begin{array}{r} \frac{dA}{dt}=k_{A}\frac{k_{m_{\alpha }}\alpha^{*}+m_{\alpha }^{o}}{\delta_{m_{\alpha }}+\delta_{B_{\alpha }}B^{2}}-\delta_{A}A \end{array}$$

(30) $$\begin{array}{r} \frac{dB}{dt}=k_{B}\frac{k_{m_{\beta }}\beta^{*}+m_{\beta }^{o}}{\delta_{m_{\beta }}+\delta_{A_{\beta }}A}-\delta_{B}B \end{array}$$

(31) $$\begin{array}{r} \frac{dS}{dt}=k_{S}\frac{k_{m_{\sigma }}\left(1-\sigma \right)+m_{\sigma }^{o}}{\delta_{m_{\sigma }}+\delta_{A\sigma }A}-\delta_{S}S \end{array}$$

## 4. Results

### 4.1. Bifurcation Analysis

**Figure 3:**
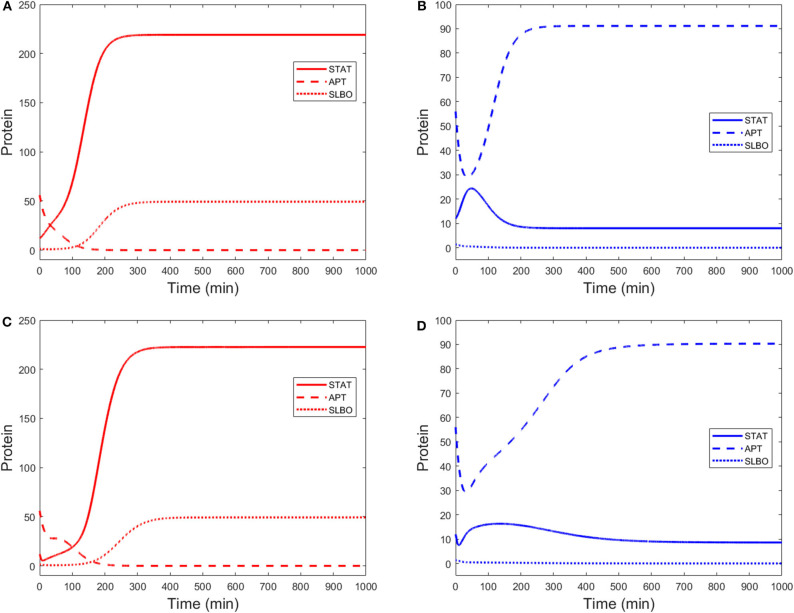
Time courses of STAT, APT, and SLBO with initial conditions STAT = 12, APT = 56, SLBO = 1.5 in the 3-variable model for **(A)** motile steady state (UPD = 4) and **(B)** stationary steady state (UPD = 1) and the 15-variable model for **(C)** motile steady state (UPD = 4) and **(D)** stationary steady state (UPD = 1).

Time courses of STAT, APT, and SLBO show the difference between the motile and stationary steady states in Figures 3A,B. Figures 3C,D shows the comparable results for the original 15-variable model. The two models converge to slightly different steady state values. This is due to the methods used to reduce the STAT dynamics in the 3-variable model. Exact steady state values can be found in the Supplementary Materials.

**Figure 4:**
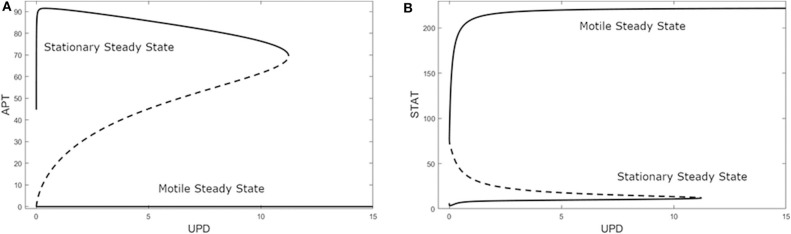
Bifurcation diagrams of **(A)** APT and **(B)** STAT against UPD in the three-variable model. A solid line indicates a stable steady state, a dashed line indicates an unstable steady state. Limit point bifurcations (where the sold and dashed lines meet) create “knees” where the model jumps from one steady state to the other. At U = 0 APT dominates.

A bifurcation diagram of APT against UPD revealed a non-trivial state when U = 0 with a high level of APT, as seen in Figure 4A. This can be interpreted as the system being predisposed to the stationary cell fate until UPD and thus STAT is high enough, at UPD >11.24 nM. As UPD approaches 0 on the upper branch, APT stops at a value of 44.73 nM. Since APT is also positively affected by STAT via UPD, very low UPD will decrease the steady state levels of APT but not to zero, leaving the stationary state the only steady state at very low UPD. Here the system encounters a limit point bifurcation and with a higher UPD level would transition to low APT and a motile cell fate.

Additionally, with the same parameters as Figure 4A, the STAT bifurcation (Figure 4B) shows that if UPD begins at a high level in a cell, the cell will remain in the motile state even as UPD decreases to a very low level. This can be seen in experiments that dissociate polar cells and border cells. The border cells continue to migrate until their UPD level presumably drops below the threshold level, i.e., as they get too far away from the polar cells (Starz-Gaiano et al., 2008; Cai et al., 2014). This threshold also exists in the opposite direction—as the level of UPD increases in an epithelial cell, it becomes a migratory border cell once the threshold is reached (Manning et al., 2015).

The bistability in the model depends on two mechanisms. We show that the predominate non-linearity is in SLBO cooperative repression of *apt* mRNA translation. We also show the repressed state for the *slbo* gene induced by APT contributes to bistability (Starz-Gaiano et al., 2008). Bifurcation diagrams for STAT, APT, and SLBO with each combination of these criteria are presented as Figures S1–S3. As quadratic non-linearity in SLBO is made linear and the ability to reach the *slbo* gene repressed state is eliminated, the stationary basin of attraction becomes smaller until bistability is lost.

### 4.2. Parameter Values

Throughout the research on parameter values, the goal was to develop a range of realistic parameters to test in our model. There are two reasons why a range of values is desirable. First, from the biological point of view, many biological processes do not occur at a constant rate. Second, heterogeneity in cellular parameters will likely lead to some parameter variety. A range of average values is thus both more appropriate and more consistent with experimental data. We were able to test the robustness of the dynamics over the ranges of parameters to see if the model behavior matches experimentally observed outcomes.

The parameter values established through research from a variety of experimental systems and testing for bistability are shown in Table 2. The range of values identified for each parameter demonstrates a robust region of bistability. This adds confidence in the decision to use some parameter values that were established from a range of possible values.

**Figure 5:**
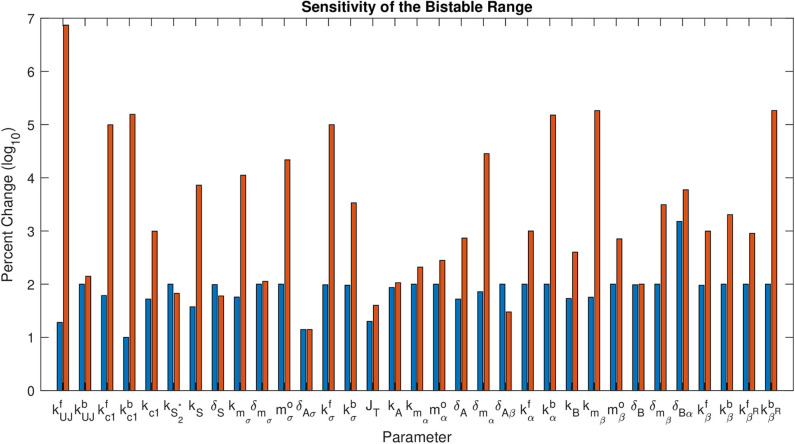
Sensitivity of the Bistable Range. This shows log_10_ of percent change in each parameter that maintains bistability. The first bar (blue) for each parameter is the negative change, the second (red) is the positive change.

Figure 5 shows the percent change on a log_10_ scale in each parameter that maintains bistability. The exact range for each parameter is listed in Table 3. The most sensitive parameter is δ_*Aσ*_. This makes sense biologically, as δ_*Aσ*_ controls APT induced miRNA degradation of *stat*. Changing this rate directly affects whether a presumptive border cell produces enough STAT activity to pass the threshold needed for motility given a particular amount of UPD. APT has multiple levels of control on STAT, but this may be the most sensitive because there are multiple other regulators at the protein level. We explore the effects of this parameter on the outcome of the system below. The range of possible values for each parameter supports the robustness of the JAK/STAT pathway as a bistable system.

**Table 3:** Bistable Range of each parameter.

**Parameter**	**Bistable start**	**Bistable end**
$k_{UJ}^{f}$	0.007	985
$k_{UJ}^{b}$	0	0.241
$k_{c_{1}}^{f}$	0.389	993
$k_{c_{1}}^{b}$	0	156
*k*_*c*_1__	47.43	1093
$k_{S_{2}^{*}}$	0	0.167
*k*_*S*_	1.875	221
δ_*S*_	0.0013	0.16
*k*_*m*_σ__	0.428	113
δ_*m*_σ__	0	2.46
$m_{\sigma }^{o}$	0	109
δ_*Aσ*_	0.043	0.057
$k_{\sigma }^{f}$	0.028	996
$k_{\sigma }^{b}$	0.089	69.6
*J*_*T*_	0.12	0.21
*k*_*A*_	0.04	0.615
*k*_*m*_α__	0	1.67
$m_{\alpha }^{o}$	0	1.65
δ_*A*_	0.019	0.334
δ_*m*_α__	0.024	24.5
δ_*A*_β__	0	0.13
$k_{\alpha }^{f}$	0	1098
$k_{\alpha }^{b}$	0	998
*k*_*B*_	0.144	1.56
*k*_*m*_β__	0.232	985
$m_{\beta }^{o}$	0	0.243
δ_*B*_	0.0011	0.08
δ_*m*_β__	0	2.77
δ_*B*_α__	0.031	30.19
$k_{\beta }^{f}$	4.7	1094
$k_{\beta }^{b}$	0	14.02
$k_{\beta^{R}}^{f}$	0	1003
$k_{\beta^{R}}^{b}$	0	959

*Any value in this range will maintain bistability in the model with other parameters held at their baseline values from Table 2*.

### 4.3. Manifolds in Three-Variable Model

We found that our three-variable minimal model retains the bistability and dynamics of the 15-variable model. Therefore, we use the three-dimensional manifolds in this model to better understand the behavior of the full model.

**Figure 6:**
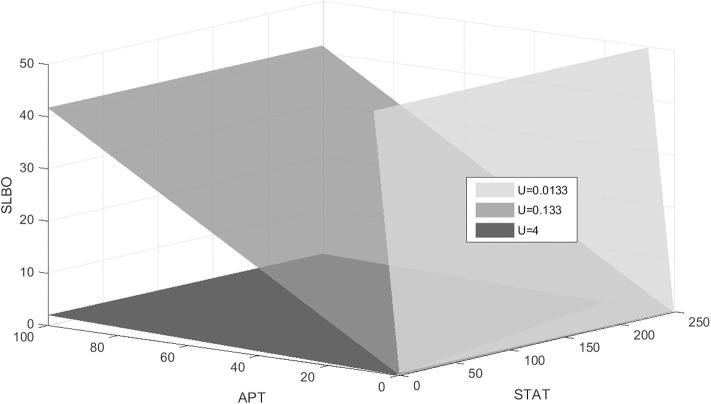
Stable manifold when UPD = 0.0133, UPD = 0.133, and UPD = 4. As UPD increases, the manifold shifts from the STAT-SLBO plane to the STAT-APT plane.

The stable manifold appears to be near-planar in the STAT-APT-SLBO phase space. The UPD value determines the position of the plane, with the manifold shifting from the STAT-SLBO plane to the STAT-APT plane as UPD increases, thus increasing STAT and SLBO production. Figure 6 shows how the manifold shifts as UPD increases from 0.0133 to 4, values that cover much of the bistable range of UPD.

**Figure 7:**
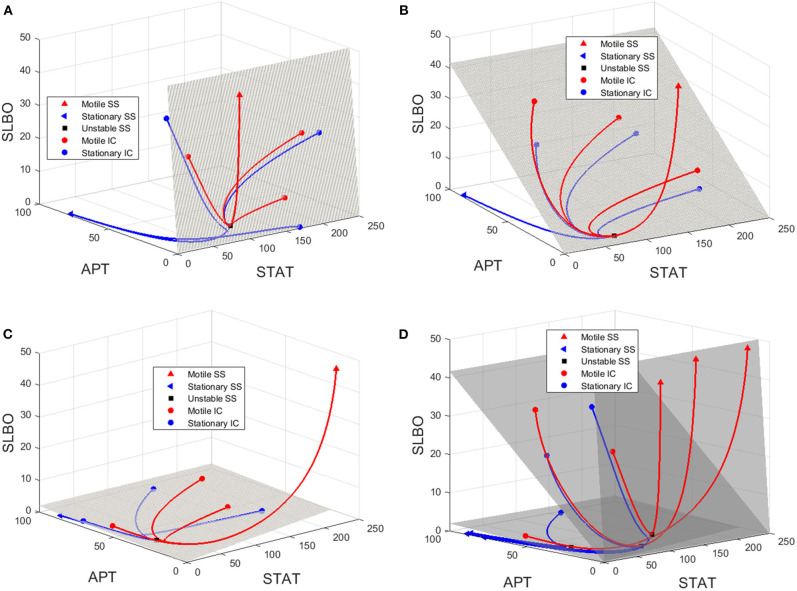
Manifolds and trajectories for three values of UPD. Stationary initial conditions and trajectories are in blue and motile initial conditions and trajectories are in red. The motile steady state is in red, the stationary steady state is in blue, and the unstable steady state is in black. **(A)** Manifold and trajectories for UPD = 0.0133. **(B)** Manifold and trajectories for UPD = 0.133. **(C)** Manifold and trajectories for UPD = 4. Movies showing a 3D rotation of each space and time series of each trajectory are available in the Figures S4-S8. **(D)** A-C combined to show how the motile basin of attraction expands as UPD increases and the manifold shifts.

To visualize the dynamics for each of the three values of UPD in the bistable region used in Figure 6, we plot trajectories to demonstrate how the manifold affects the outcomes of different initial conditions. Initial conditions that lie just below the manifold are attracted to the stable manifold near the unstable steady state and then repelled toward the stationary steady state. Initial conditions that lie just above the manifold behave similarly but converge to the motile steady state. Figure 7 depicts this behavior, with initial conditions that progress to the stationary steady state and the corresponding trajectories in blue and initial conditions that progress to the motile steady state and the corresponding trajectories in red. Three initial conditions and trajectories for each steady state are plotted. The motile steady state is in red, the stationary steady state is in blue, and the unstable steady state is in black. The unstable steady state lies on the 2D stable manifold.

When UPD is low, such as in Figure 7A, initial conditions in most of the phase space will result in the stationary steady state. When UPD is high, such as in Figure 7C, initial conditions in most of the phase space will result in the motile steady state.

Figure 7D combines Figures 7A–C to show how the shifting manifold affects the system. As the value of UPD increases and the manifold moves below additional initial conditions, the motile basin of attraction expands and the motile and stationary steady states shift slightly. Movies showing a 3D rotation of each space and time series of each trajectory are available in the Figures S4–S8.

### 4.4. Delay From miRNA

**Figure 8:**
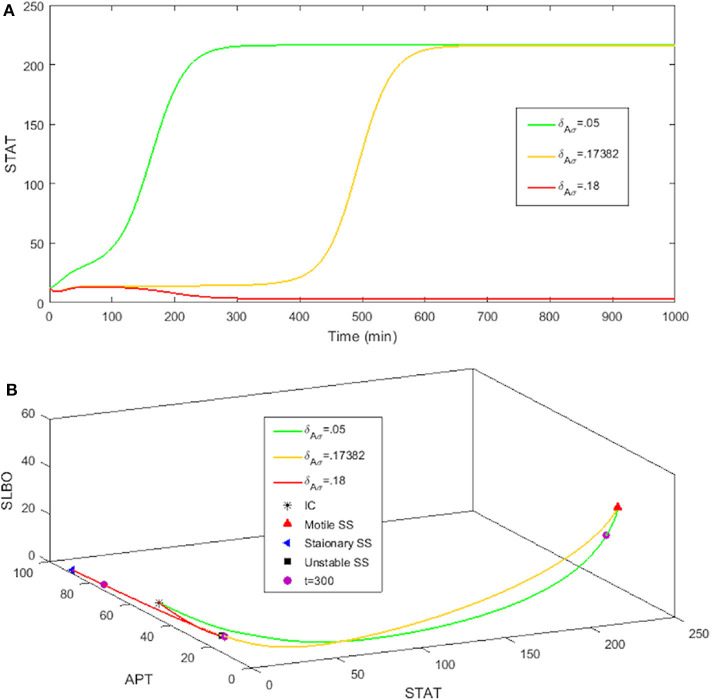
Three levels of miRNA controlled by δ_*Aσ*_: GO δ_*Aσ*_ = 0.05, STOP δ_*Aσ*_ = 0.18, SLOW δ_*Aσ*_ = 0.17382 with initial conditions STAT = 12, APT = 56, SLBO = 1.5, and UPD = 4. **(A)** Time courses of STAT. As δ_*Aσ*_ increases STAT activation is delayed. **(B)** 3D plot of the three cell fates in **(A)**. The motile steady state is in red, the stationary steady state is in blue, and the unstable steady state is in black. *t* = 300 on each trajectory is marked in purple.

The rate at which APT-induced miRNAs lead to degradation of *stat* mRNA is controlled in the model by the parameter δ_*Aσ*_. Figure 8A shows time courses of *S* in the three-variable model with UPD = 4 for different values of δ_*Aσ*_. We established a typical value of δ_*Aσ*_ = 0.05 that allows *S* to equilibrate around 200 min, which is consistent with the time it takes for border cells to respond to UPD (Starz-Gaiano et al., 2008; Manning et al., 2015). The STAT level when δ_*Aσ*_ = 0.05 is well above the threshold needed for the cell to become motile. An increase to δ_*Aσ*_ = 0.17382 delays STAT convergence by a significant amount of time, about 600 min. If we increase δ_*Aσ*_ to just 0.18, *S* never elevates within 1,000 min. Thus, the cell remains stationary. Each time course in Figure 8 represents a different outcome of the JAK/STAT pathway: GO, where SLBO dominates (δ_*Aσ*_ = 0.05); STOP, where APT dominates (δ_*Aσ*_ = 0.18); and SLOW, where the transition to motility is delayed (δ_*Aσ*_ = 0.17382). The mechanisms of how APT-activated miRNAs affect STAT are still being analyzed, but initial findings support the idea that changes in miRNA activity can cause delays similar to those seen in our model (Yoon et al., 2011; Luo and Sehgal., 2012; Monahan and Starz-Gaiano, 2016; Sun et al., 2015). Delays in STAT activation and failure of activation are possible within a realistic time frame.

**Figure 9:**
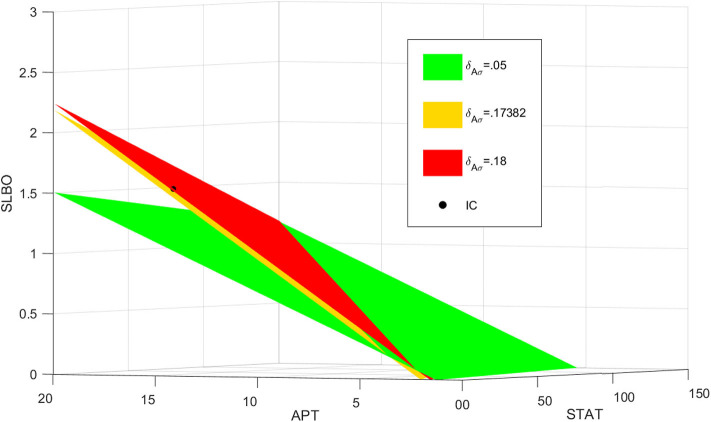
Manifolds for $\delta_{\text{A}\sigma }=0.05$, $\delta_{\text{A}\sigma }=0.18$, $\delta_{\text{A}\sigma }=0.17382$. As δ_*Aσ*_ increases, the manifold shifts above the initial condition in black.

For basal levels of δ_*Aσ*_ the system has normal STAT activation. However, as δ_*Aσ*_ is increased the manifold separating the GO and STOP basins of attraction gets close to the initial condition, slowing the rise time to activation. If δ_*Aσ*_ is raised enough the manifold crosses the initial condition and the trajectory is attracted to the STOP fate. Figure 8B further illustrates this delay. The 3D plot shows that at *t* = 300 the SLOW cell fate still has very low STAT and SLBO production. At the same time the GO and STOP cell fates have almost converged to their respective steady states. This change in outcome for one initial condition occurs because the increase in δ_*Aσ*_ causes the manifold to shift just above the initial condition. This can be seen in Figure 9, where the initial condition lies between the SLOW manifold and the STOP manifold.

### 4.5. Experimental Tests

We tested our models to confirm if they reproduced certain behaviors identified in various experiments. It has been shown that if STAT activity is blocked by stage 9 of cell migration, the level of APT protein drops by about half (Starz-Gaiano et al., 2008, 2009; Yoon et al., 2011; Monahan and Starz-Gaiano, 2015). It is also known that increasing the initial condition of APT should decrease the level of STAT protein and activity (Starz-Gaiano et al., 2008). These behaviors should be achievable by our models.

**Figure 10:**
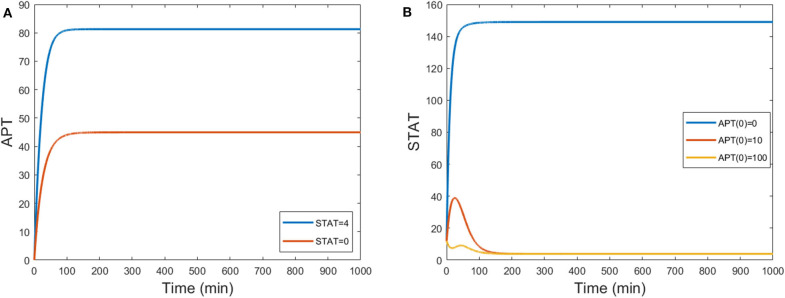
**(A)** Time courses of APT show levels decrease by half when STAT is knocked down from a steady state value of around 4 to one of 0 by setting *k*_*S*_ = 0. **(B)** Time courses of STAT for different initial conditions of APT. When UPD = 0.1, STAT steady state levels decrease for high initial conditions of APT. S(0) = 12 and B(0) = 1.5.

The three-variable model (Equations 29–31) reproduces the experimental behavior of APT when STAT is knocked down, as shown in Figure 10A. STAT initially converges to a value of about 4. Setting *k*_*S*_ = 0 causes STAT to be constantly zero. This causes APT to converge to an equilibrium roughly half that of the stationary steady state. APT acts as an inhibitor to STAT activity, so for higher APT initial conditions we should see lower levels of STAT. This also occurs in the three-variable model (Figure 10B).

### 4.6. Comparison to Previous Models

Previous mathematical models of the JAK/STAT pathway in *Drosophila* have focused on matching the qualitative behavior of the system. We compare our mechanistic model to the models developed in Starz-Gaiano et al. (2008) and Yoon et al. (2011) in Table 4. We rewrite our equations and use composite parameters to allow for easier comparison. The names of variables for STAT and SLBO differ across the models.

**Table 4:** Comparison of models.

**Variable**	**Starz-Gaiano et al. (2008)**	**Yoon et al. (2011)**	**This paper**
JAK/STAT	$\frac{dJ}{dt}=\frac{0.04U}{1+10P^{2}+0.1J^{2}+\frac{33A^{2}}{1+15S}}-0.01J$	$\frac{dJ}{dt}=\frac{p_{j}U}{1+q_{j}P^{2}+\frac{a_{j}R}{1+k_{j}S^{2}}}-r_{j}J$	$\frac{dS}{dt}=\frac{k_{S}k_{m_{\sigma }}\frac{S^{2}}{S^{2}+k_{m}^{2}}\frac{U}{U+K_{UJ}}+m_{\sigma }^{o}}{\left(\delta_{1}+A\right)\left[\frac{K_{\sigma }k_{S_{2}^{*}}}{k_{c_{1}}J_{T}}+\frac{S^{2}}{S^{2}+k_{m}^{2}}\frac{U}{U+K_{UJ}}\right]}-\delta_{S}S$
UPD	$\frac{dU}{dt}=0.01P-0.006U+0.1\frac{d^{2}U}{dx^{2}}$	$\frac{dU}{dt}=p_{u}P-r_{u}U+d_{u}\frac{d^{2}U}{dx^{2}}$	U is a parameter.
APT	$\frac{dA}{dt}=\frac{0.0001J}{1+4A^{2}+S}-0.0001A+\text{1e-5}$	$\frac{dA}{dt}=\frac{p_{a}J}{1+q_{a}S}-r_{a}A+b_{a}$	$\frac{dA}{dt}=\frac{\hat{k_{1}}}{\left(\delta_{2}+B^{2}\right)\frac{S^{2}}{S^{2}+k_{m}^{2}}\frac{U}{U+K_{UJ}}}-\delta_{A}A+\frac{K_{1}}{\delta_{2}+B^{2}}$
SLBO	$\frac{dS}{dt}=\frac{0.002J^{2}}{1+0.5A^{2}}-0.0005S$	$\frac{dS}{dt}=\frac{p_{s}J^{2}}{1+q_{s}A}-r_{s}S$	$\frac{dB}{dt}=\frac{\hat{k_{2}}\hat{k_{3}}\frac{S^{2}}{S^{2}+k_{m}^{2}}\frac{U}{U+K_{UJ}}}{\left(\delta_{3}+A\right)\left(1+\hat{k_{3}}\frac{S^{2}}{S^{2}+k_{m}^{2}}\frac{U}{U+K_{UJ}}+\frac{A}{K_{\beta^{R}}}\right)}-\delta_{B}B+\frac{K_{2}}{\delta_{3}+A}$
miRNA279		$\frac{dR}{dt}=\frac{p_{r}A}{1+q_{r}K^{2}}-r_{r}R$	
Ken		$\frac{dK}{dt}=p_{k}J-r_{k}K$	

*Composite parameters:* $K_{UJ}=\frac{k_{UJ}^{b}}{k_{UJ}^{f}},\delta_{1}=\frac{\delta_{m_{\sigma }}}{\delta_{A\sigma }},$

$\hat{k_{1}}=\frac{k_{A}k_{m_{\alpha }}K_{\alpha }k_{S_{2}^{*}}}{\delta_{B\alpha }k_{c_{1}}J_{T}},K_{1}=\frac{k_{A}m_{\alpha }^{o}}{\delta_{B\alpha }},\delta_{2}=\frac{\delta_{m_{\alpha }}}{\delta_{B\alpha }},\hat{k_{2}}=\frac{k_{B}k_{m_{\beta }}}{\delta_{A\beta }},\hat{k_{3}}=\frac{k_{c_{1}}J_{T}}{k_{S_{2}^{*}}K_{\beta }},K_{2}=\frac{k_{B}m_{\beta }^{o}}{\delta_{A\beta }},\delta_{3}=\frac{\delta_{m_{\beta }}}{\delta_{A\beta }}$.

Our model and the earlier models share the same basic structure. The equations for STAT, APT, and SLBO all have a production term and a degradation term. The production terms include the cross-reactions of the proteins. However, our model describes these interactions in more detail, accounting for more of the known molecular interactions and biochemical production/degradation rates. For example, our STAT equation includes the feedback inhibition from APT. Our model also includes STAT independent production rates for both APT and SLBO, while the earlier models only include this for APT. By including the details of these molecular interactions, we gain the ability to mathematically examine the bistability of the JAK/STAT pathway.

Dynamic UPD is a feature of earlier models. Earlier models also include that levels of active STAT are positively regulated by production of UPD. Our model can be combined with the UPD dynamics developed in Manning et al. (2015). They used a partial differential equation to model the change in UPD concentration over time in three-dimensional extracellular space (Manning et al., 2015). This more detailed version of UPD dynamics includes the spatial element of how UPD diffuses from the polar cells into the border cells. It also helps to explain how border cells farther from the polar cells, which receive less UPD, can be delayed in becoming motile. Additionally, modeling in Peercy and Starz-Gaiano (2020) gives details of how the geometry of the egg chamber influences cell fate and migration dynamics. They also examine models of velocity and migratory cohort size as well as how clusters of border cells function to guide directional movement. Together our mechanistic model, the spatial dynamics of UPD from Manning et al. (2015), and models of collective migration from Peercy and Starz-Gaiano (2020) help us gain a better understanding of the threshold for motility in border cells.

## 5. Discussion

We reduced the mechanistic Ge and Stonko model in stages to arrive at our minimal three-variable model. This final model allows us to better understand bistability in the JAK/STAT pathway. Our minimal model retains the parameters from the more complex model, allowing easier analysis but retaining critical properties. We discovered that non-linearity in SLBO repression of *apt* mRNA translation (Equation 9), and to a lesser extent APT repression of the *slbo* gene is required for bistability. The model displays the bistability of the stationary and motile cell states expected from experimental data for medium saturation of STAT activity. It has been shown that modulating UPD expression affects the number of migratory cells (Manning et al., 2015; Van De Bor et al., 2011; Silver and Montell, 2001; Xi et al., 2003; Grammont and Irvine, 2002). If UPD is low there are no migratory cells and if UPD is high there are many. This can induce normally stationary cells far away from the anterior to become motile (Manning et al., 2015).

We established parameters that showed bistability was obtainable under realistic conditions. Every parameter in the 15-variable model was found to have a wide range of values that guaranteed bistability. The robustness with respect to parameter values also suggests the biophysical utility of the model. Cell migration is an essential biological process, so the JAK/STAT pathway necessitates robustness under a range of parameter values, which the model supports. It makes sense that the JAK/STAT pathway would be able to operate successfully under a range of parameter values.

Ge and Stonko assumed that a key aspect necessary for bistability in APT and SLBO is cooperativity in SLBO repressing *apt* mRNA. In our dynamic STAT model, we found that the feedback inhibition of APT on STAT is necessary for bistability in STAT activity coming from the bistability between APT and SLBO. We also found that the initial amount of APT present in a cell is a major factor in whether or not the cell will become motile through affecting the level of STAT activity. Our minimal reduced model also reproduces behavior seen in experiments, such as declines in APT expression when STAT is knocked down and how initial levels of APT affect final levels of STAT (Starz-Gaiano et al., 2008).

We also showed that delays in STAT activation and failure of activation are possible within a realistic time frame. By controlling the degree of feedback inhibition of APT on STAT we can induce a delay in the transition to cell motility or cause the cell to remain stationary. This result is due to the proximity of the UPD level to a limit point bifurcation.

Assumptions made in the development of this model lead to some limitations. Specific parameter values for the exact reactions occurring in the egg chamber are not known, so we performed sensitivity analysis to show the robustness in parameter values. The STAT dynamics were reduced under a number of assumptions. One was the decision to ignore the *c*_2_ equation, which allows APT to act as a buffer on STAT. Since the action of APT on STAT is known to function by preventing activation through miRNAs (Yoon et al., 2011) as well as limiting active protein present (Monahan and Starz-Gaiano, 2015, 2016), this assumption might oversimplify the larger system. Additionally, the quasi-steady state assumptions made in reducing STAT dynamics may also oversimplify the model.

Further study into the various methods by which APT inhibits STAT would enable us to improve how the model captures this interaction. Experiments that change the level of APT or STAT may give more information about how the delay in specification affects the migration of the cell cluster. Experimenting with *apt* mutants could tell us more about how the bistability we show in cell specification ultimately affects cluster migration and the further development of the egg chamber. When STAT is abnormally high more intermediate border cells trail behind the polar cells (Silver and Montell, 2001; Silver et al., 2005; Monahan and Starz-Gaiano, 2015; Starz-Gaiano et al., 2008). Socs36E limits STAT signaling and higher Socs36E reduces motile cell number. Both of these cases may suggest how a delay in cell specification affects cell migration.

It may also be important to consider how APT and SLBO may interact through miRNAs. Currently there is little data beyond the existence of these miRNAs (Ge et al., 2012). miRNA interactions directly affect bistability in the model, so greater detail could improve the model. Experiments that control the level of UPD secreted could identify how quickly the signal enters border cells and the levels of UPD which correspond with the activated STAT threshold to induce motility. To further gauge the STAT levels it would also be valuable to know the level of APT present in border cells through activation by EYA, prior to STAT activation and upregulation by STAT.

The JAK/STAT signaling pathway is known to be well-conserved. Specifically, it seems to be comparable in *Drosophila* and in humans (Trivedi and Starz-Gaiano, 2018; Arbouzova and Zeidler, 2006; Amoyel and Bach, 2012; Amoyel et al., 2014). Thus, as the model we have developed helps to explain cell motility in *Drosophila*, it may prove useful to our understanding of the process in humans. The “decision” for tumor cells to become motile is often a turning point for cancer progression. Additionally, STAT signaling is also well-known for controlling stem cell division decisions. Thus, the decisiveness of STAT-based signaling may have a variety of roles in different cell types, but the model and its bistability shown in this paper may help to explain how this STAT signaling operates in different situations.

## Data Availability Statement

The raw data supporting the conclusions of this article will be made available by the authors, without undue reservation.

## Author Contributions

BP and MS-G contributed conception and methodology, and reviewed and edited the manuscript. AB was the main writer the manuscript, and developed the reduced model, performed analysis, and ran simulations. All authors contributed to manuscript revision, read, and approved the submitted version.

## Conflict of Interest

The authors declare that the research was conducted in the absence of any commercial or financial relationships that could be construed as a potential conflict of interest.

## References

[B1] Aman A. Piotrowski T. (2010). Cell migration during morphogenesis. Dev. Biol. 341, 20–33. 10.1016/j.ydbio.2009.11.014 19914236

[B2] Amoyel M. Anderson A. M. Bach E. A. (2014). JAK/STAT pathway dysregulation in tumors: a Drosophila perspective. Semin. Cell Dev. Biol. 28, 96–103. 10.1016/j.semcdb.2014.03.023 24685611 PMC4037387

[B3] Amoyel M. Bach E. A. (2012). Functions of the Drosophila JAK-STAT pathway: lessons from stem cells. Jak Stat 1, 176–183. 10.4161/jkst.21621 24058767 PMC3670241

[B4] Arbouzova N. I. Zeidler M. P. (2006). JAK/STAT signalling in Drosophila: insights into conserved regulatory and cellular functions. Development 133, 2605–2616. 10.1242/dev.02411 16794031

[B5] Betel D. Wilson M. Gabow A. Marks D. S. Sander C. (2007). The microRNA.org resource: targets and expression. Nucleic. Acids. Res. 36, 149–153. 10.1093/nar/gkm995 18158296 PMC2238905

[B6] Cai D. Chen S.-C. Prasad M. He L. Wang X. Choesmel-Cadamuro V.. (2014). Mechanical feedback through E-cadherin promotes direction sensing during collective cell migration. Cell 157, 1146–159. 10.1016/j.cell.2014.03.045 24855950 PMC4118667

[B7] Chen Y. Kotian N. Aranjuez G. Chen L. Messer C. L. Burtscher A.. (2020). Protein phosphatase 1 activity controls a balance between collective and single cell modes of migration. Elife 9:e52979. 10.7554/eLife.52979.sa2 32369438 PMC7200163

[B8] Ekas L. A. Cardozo T. J. Flaherty M. S. McMillan E. A. Gonsalves F. C. Bach E. A. (2010). Characterization of a dominant-active stat that promotes tumorigenesis in Drosophila. Dev. Biol. 344, 621–636. 10.1016/j.ydbio.2010.05.497 20501334 PMC2914209

[B9] Ermentrout B. (2002). Simulating, Analyzing, and Animating Dynamical Systems: A Guide to XPPAUT for Researchers and Students. Philadelphia, PA: Society for Industrial and Applied Mathematics.

[B10] Friedl P. Mayor R. (2017). Tuning collective cell migration by cell–cell junction regulation. Cold Spring Harbor Perspect. Biol. 9:a029199. 10.1101/cshperspect.a029199 28096261 PMC5378050

[B11] Ge X. Stonko D. Peercy B. Starz-Gaiano M. (2012). Modelling a cellular response to a gradient. UMBC Rev. 13, 92–113.

[B12] Ghiglione C. Devergne O. Georgenthum E. Carballès F. Médioni C. Cerezo D.. (2002). The Drosophila cytokine receptor Domeless controls border cell migration and epithelial polarization during oogenesis. Development 129, 5437–5447. 10.1242/dev.00116 12403714

[B13] Grammont M. Irvine K. D. (2002). Organizer activity of the polar cells during *Drosophila* oogenesis. Development 129, 5131–5140. Available online at: https://dev.biologists.org/content/129/22/5131 12399305 10.1242/dev.129.22.5131

[B14] Guido N. J. Wang X. Adalsteinsson D. McMillen D. Hasty J. Cantor C. R.. (2006). A bottom-up approach to gene regulation. Nature 439, 856–860. 10.1038/nature04473 16482159

[B15] Halford S. E. Marko J. F. (2004). How do site-specific DNA-binding proteins find their targets? Nucleic Acids Res. 32, 3040–3052. 10.1093/nar/gkh624 15178741 PMC434431

[B16] Hargrove J. L. Hulsey M. G. Beale E. G. (1991). The kinetics of mammalian gene expression. Bioessays 13, 667–674. 10.1002/bies.950131209 1789784

[B17] Harris R. E. Pargett M. Sutcliffe C. Umulis D. Ashe H. L. (2011). Brat promotes stem cell differentiation via control of a bistable switch that restricts BMP signaling. Dev. Cell 20, 72–83. 10.1016/j.devcel.2010.11.019 21238926 PMC3178012

[B18] Hilton D. Nicola N. (1992). Kinetic analyses of the binding of leukemia inhibitory factor to receptor on cells and membranes and in detergent solution. J. Biol. Chem. 267, 10238–10247. 1587813

[B19] Karsten P. Plischke I. Perrimon N. Zeidler M. P. (2006). Mutational analysis reveals separable DNA binding and trans-activation of Drosophila STAT92E. Cell. Signal. 18, 819–829. 10.1016/j.cellsig.2005.07.006 16129580

[B20] Leonard C. E. Taneyhill L. A. (2020). The road best traveled: neural crest migration upon the extracellular matrix. Semin. Cell Dev. Biol. 100, 177–185. 10.1016/j.semcdb.2019.10.013 31727473 PMC7071992

[B21] Lewin B. (2004). Genes VIII. Upper Saddle River, NJ: Pearson Prentice Hall.

[B22] Lui P.-Y. Jin D.-Y. Stevenson N. J. (2015). MicroRNA: master controllers of intracellular signaling pathways. Cell. Mol. Life Sci. 72, 3531–3542. 10.1007/s00018-015-1940-0 26059472 PMC11113591

[B23] Luo W. Sehgal A. (2012). Regulation of circadian behavioral output via a MicroRNA-JAK/STAT circuit. Cell 148, 765–779. 10.1016/j.cell.2011.12.024 22305007 PMC3307393

[B24] Manning L. A. Weideman A. M. Peercy B. E. Starz-Gaiano M. (2015). Tissue landscape alters adjacent cell fates during Drosophila egg development. Nat. Commun. 6:7356. 10.1038/ncomms8356 26082073 PMC4473798

[B25] McGregor J. R. Xi R. Harrison D. A. (2002). JAK signaling is somatically required for follicle cell differentiation in Drosophila. Development 129, 705–717. Available online at: https://dev.biologists.org/content/129/3/705 11830571 10.1242/dev.129.3.705

[B26] Monahan A. Starz-Gaiano M. (2016). Apontic regulates somatic stem cell numbers in Drosophila testes. BMC Dev. Biol. 16:5. 10.1186/s12861-016-0103-3 26993259 PMC4799534

[B27] Monahan A. J. Starz-Gaiano M. (2015). Socs36E limits STAT signaling via Cullin2 and a SOCS-box independent mechanism in the Drosophila egg chamber. Mech. Dev. 138(Pt 3), 313–327. 10.1016/j.mod.2015.08.003 26277564

[B28] Montell D. J. Rorth P. Spradling A. C. (1992). Slow border cells, a locus required for a developmentally regulated cell migration during oogenesis, encodes Drosophila C/EBP. Cell 71, 51–62. 10.1016/0092-8674(92)90265-E 1394432

[B29] Montell D. J. Yoon W. H. Starz-Gaiano M. (2012). Group choreography: mechanisms orchestrating the collective movement of border cells. Nat. Rev. Mol. Cell Biol. 13, 631–645. 10.1038/nrm3433 23000794 PMC4099007

[B30] Nicholson S. E. Nicola N. A. (eds.). (2013). JAK-STAT Signalling: Methods and Protocols, Volume 967 of Methods in Molecular Biology. New York, NY: Humana Press.

[B31] Olson H. M. Nechiporuk A. V. (2018). Using zebrafish to study collective cell migration in development and disease. Front. Cell Dev. Biol. 6:83. 10.3389/fcell.2018.00083 30175096 PMC6107837

[B32] Parsaeian A. de la Cruz M. O. Marko J. F. (2013). Binding-rebinding dynamics of proteins interacting non-specifically with a long DNA molecule. Phys. Rev. E Stat Nonlinear Soft Matter Phys. 4:040703. 10.1103/PhysRevE.88.040703 24229102 PMC3894571

[B33] Peercy B. E. Starz-Gaiano M. (2020). Clustered cell migration: modeling the model system of Drosophila border cells. Semin. Cell Dev. Biol. 100, 167–176. 10.1016/j.semcdb.2019.11.010 31837934

[B34] Rorth P. (1994). Specification of C/EBP function during Drosophila development by the bZIP basic region. Science 266, 1878–1881. 10.1126/science.7997882 7997882

[B35] Rorth P. Szabo K. Texido G. (2000). The level of C/EBP protein is critical for cell migration during Drosophila oogenesis and is tightly controlled by regulated degradation. Mol. Cell 6, 23–30. 10.1016/S1097-2765(05)00008-0 10949024

[B36] Saadin A. Starz-Gaiano M. (2016). Circuitous genetic regulation governs a straightforward cell migration. Trends Genet. 32, 660–673. 10.1016/j.tig.2016.08.001 27600524

[B37] Silver D. L. Geisbrecht E. R. Montell D. J. (2005). Requirement for JAK/STAT signaling throughout border cell migration in Drosophila. Development 132, 3483–3492. 10.1242/dev.01910 16000386

[B38] Silver D. L. Montell D. J. (2001). Paracrine signaling through the JAK/STAT pathway activates invasive behavior of ovarian epithelial cells in Drosophila. Cell 107, 831–841. 10.1016/S0092-8674(01)00607-9 11779460

[B39] Starz-Gaiano M. Melani M. Meinhardt H. Montell D. (2009). Interpretation of the UPD/JAK/STAT morphogen gradient in Drosophila follicle cells. Cell Cycle 8, 2917–2925. 10.4161/cc.8.18.9547 19729999 PMC3021920

[B40] Starz-Gaiano M. Melani M. Wang X. Meinhardt H. Montell D. J. (2008). Feedback inhibition of JAK/STAT signaling by apontic is required to limit an invasive cell population. Dev. Cell 14, 726–738. 10.1016/j.devcel.2008.03.005 18477455

[B41] Stuelten C. H. Parent C. A. Montell D. J. (2018). Cell motility in cancer invasion and metastasis: insights from simple model organisms. Nat. Rev. Cancer 18, 296–312. 10.1038/nrc.2018.15 29546880 PMC6790333

[B42] Sun K. Jee D. de Navas L. F. Duan H. Lai E. C. (2015). Multiple *in vivo* biological processes are mediated by functionally redundant activities of Drosophila mir-279 and mir-996. PLoS Genet. 11:e1005245. 10.1371/journal.pgen.1005245 26042831 PMC4456407

[B43] Thurmond J. Goodman J. Strelets V. Attrill H. Gramates L. Marygold S.. (2019). Flybase 2.0: the next generation. Nucleic Acids Res. 47, D759–D765. 10.1093/nar/gky1003 30364959 PMC6323960

[B44] Trivedi S. Starz-Gaiano M. (2018). Drosophila Jak/STAT signaling: regulation and relevance in human cancer and metastasis. Int. J. Mol. Sci. 19:4056. 10.3390/ijms19124056 30558204 PMC6320922

[B45] Van De Bor V. Zimniak G. Cerezo D. Schaub S. Noselli S. (2011). Asymmetric localisation of cytokine mRNA is essential for JAK/STAT activation during cell invasiveness. Development 138, 1383–1393. 10.1242/dev.056184 21350010

[B46] Ward L. Howlett G. Hammacher A. (1995). Use of a biosensor with surface plasmon resonance detection for the determination of binding constants: measurement of interleukin-6 binding to the soluble interleukin-6 receptor. Biochemistry 34, 2901–2907. 10.1021/bi00009a021 7893704

[B47] Wright V. M. Vogt K. L. Smythe E. Zeidler M. P. (2011). Differential activities of the Drosophila JAK/STAT pathway ligands Upd, Upd2 and Upd3. Cell. Signal. 23, 920–927. 10.1016/j.cellsig.2011.01.020 21262354

[B48] Xi R. McGregor J. R. Harrison D. A. (2003). A gradient of JAK pathway activity patterns the anterior-posterior axis of the follicular epithelium. Dev. Cell 4, 167–177. 10.1016/S1534-5807(02)00412-4 12586061

[B49] Yang E. Henriksen M. A. Schaefer O. Zakharova N. Darnell J. E. (2002). Dissociation time from DNA determines transcriptional function in a STAT1 linker mutant. J. Biol. Chem. 277, 13455–13462. 10.1074/jbc.M112038200 11834743

[B50] Yoon W. H. Meinhardt H. Montell D. J. (2011). miRNA-mediated feedback inhibition of JAK/STAT morphogen signalling establishes a cell fate threshold. Nat. Cell Biol. 13, 1062–1069. 10.1038/ncb2316 21857668 PMC3167036

