# Supplementary material for: Development and Analysis of a Quantitative Mathematical Model of Bistability in the Cross Repression System Between APT and SLBO Within the JAK/STAT Signaling Pathway
Source: Front Physiol. 2020 Jul 28;11:803. doi: 10.3389/fphys.2020.00803 (PMC7401978; doi:10.3389/fphys.2020.00803)
Supplement: Supplementary file 1 [file Data_Sheet_1.PDF]

Supplementary Material

1 SUPPLEMENTARY DATA

1.1 Steady State Values

The steady state values for the 15-variable model, equations (1)-(15), are listed in Table S1 and the values for the three-variable model, equations (29)-(31), are listed in Table S2. A small change in the steady state values occurs due to the reductions made.

1.2 XPP Code

XPP code for Ge and Stonko's 15-variable model:

```
# Unpaired value
par U=4

# parameters
p kfUJ=.0133, kbUJ=0.1, kfc1=1, kbc1=0.1, kc1=100, kSstar=0.10, ks=3,
p ds=.1, kfSA=0.01, kbSA=10, dA=.04, dB=.04, kA=0.298, kB=.312, kma=.54,
p kmb=.538, kms= 1, dma=0.086, dmb=.086, mao=0.52, mbo=0.01, mso=0.5,
p dBa=0.5, dAb=0.1, dAs=0.05, kfa=100, kba=.66, kfb=100, kbb=.66,
p kfbr=100, kbbr=0.522, kfs=1, kbs=2, JT=0.15, dms =.2

# initial values
J_15(0) = 0.15

# equations
ma_15'=kma*(1-alph_15)-dma*ma_15+mao-dBa*(B_15)^2*ma_15

Jstar_15'=kfUJ*U*J_15-kbUJ*Jstar_15-kfc1*Jstar_15*(S_15)^2+kbc1*c1_15
+kc1*c1_15

J_15'=-kfUJ*U*J_15 + kbUJ * Jstar_15

S_15'=-2 * kfc1 * Jstar_15 * (S_15)^2+2 * kbc1 * c1_15 +
2 * kSstar * Sstar_15 + kS * ms_15 - dS * S_15

c1_15'=kfc1 * Jstar_15 * (S_15)^2 - kbc1 * c1_15 - kc1 * c1_15

c2_15'=kfSA * Sstar_15 * A_15 - kbSA * c2_15

Sstar_15'=kc1 * c1_15 - kSstar * Sstar_15- kfSA * Sstar_15 * A_15
+ kbSA * c2_15

A_15'=kA * ma_15 - dA * A_15 - kfSA * Sstar_15 * A_15 + kbSA *c2_15
```

```
B_15'=kB * mb_15 - dB * B_15

mb_15'=kmb * (1-bet_15-bR_15) - dmb * mb_15 + mbo - dAb * A_15 * mb_15

alph_15'=-kfa * Sstar_15 * alph_15 + kba * (1-alph_15)

bet_15'=-kfb * Sstar_15 * bet_15 + kbb * (1-bet_15-bR_15)
- kfbr * A_15 * bet_15 + kbbr * bR_15

bR_15'=kfbr * A_15 * bet_15 - kbbr * bR_15

sig_15'=-kfs * Sstar_15 * sig_15 + kbs *(1-sig_15)

ms_15'=kms *(1-sig_15) - dms * ms_15 + mso - dAs * A_15 * ms_15

@ total=1000
@ xlo=0 xhi=1000 ylo=0 yhi=250
@ method=cvode, bounds=10000000, total=1000, maxstor=10000001
@ nmax=2000, npr=5000, parmax=20, autovar=S_15

done
```

XPP code for the reduced 3-variable model:

```
# Unpaired value
p U=4

# parameters
p kfUJ=.0133, kbUJ=0.1, kfc1=1, kbc1=0.1, kc1=100, kSstar=0.10, ks=3,
p ds=.1, kfSA=0.01, kbSA=10, dA=.04, dB=.04, kA=0.298, kB=.312,
p kma=.54, kmb=.538, kms=1, dma=0.086, dmb=.086, mao=0.52, mbo=0.01,
p mso=0.5, dBa=0.5, dAb=0.1, dAs=0.05, kfa=100, kba=.66, kfb=100,
p kbb=.66, kfbr=100, kbbr=0.522, kfs=1, kbs=2, dms=.2, jtotal=.15

# quasi-steady state approximations
Jstar=u/(u+kbUJ/kfUJ)*jtotal

vmax=kc1*Jstar

kmsq=(kbc1+kc1)/kfc1

# equations
dS/dt=kS*(kms*(1-1/(1+(vmax*(S)^2/((S)^2+kmsq))/kSstar*kfs/kbs))+mso)
/(dms+dAs*A)-dS*S

dA/dt=kA*((kma*(1-kba/((kfa*(vmax*(S)^2/((S)^2+kmsq))/kSstar)+kba))
```

```
+mao)/(dma+dba*(b^2)))-dA*A

dB/dt=kB*((kmb*(((vmax*(S)^2/((S)^2+kmsq))/kSstar*kfb/kbb))
/(((vmax*(S)^2/((S)^2+kmsq))/kSstar*kfb/kbb)+1+((A*kfbr/kbbr)))+mbo)
/(dmb+dab*A))-dB*B

@ total=1000
@ meth=stiff, bounds=10000000, maxstor=1000000
@ xlo=0, xhi=1000, ylo=0, yhi=250
@ nmax=2000, npr=5000, parmax=20
done
```

2 SUPPLEMENTARY TABLES AND FIGURES

2.1 Tables

Variable	Steady State 1 (SS1): Motile (Red)	Steady State 2 (SS2): Stationary (Blue)
J^*	0.00024	0.0026
J	0.034	0.147
S	221.3	4.43
c_1	0.115	0.00051
c_2	0.00074	0.046
S_2^*	115.13	0.510
A	0.0065	91.17
B	49.33	0.0104
m_α	0.00087	12.238
m_β	6.324	0.0013
m_σ	7.40	0.148
α	0.00005	0.013
β	0.000004	0.00006
β^R	0.000007	0.996
σ	0.017	0.797

Table S1. Steady state values with $U = 0.133$ in the 15-variable model, equations (1)-(15).

Variable	Steady State 1 (SS1): Motile (Red)	Steady State 2 (SS2): Stationary (Blue)
S	159.479	4.185
A	0.0065	90.99
B	49.05	0.01002

Table S2. Steady state values with $U = 0.133$ in the three-variable model, equations (29)-(31).

2.2 Figures

Bifurcation diagrams for STAT, APT, and SLBO as referenced in Section 4.1. Illustrates loss of bistability caused by losing non-linearity in SLBO repressing APT and combining β and β^R into one variable.

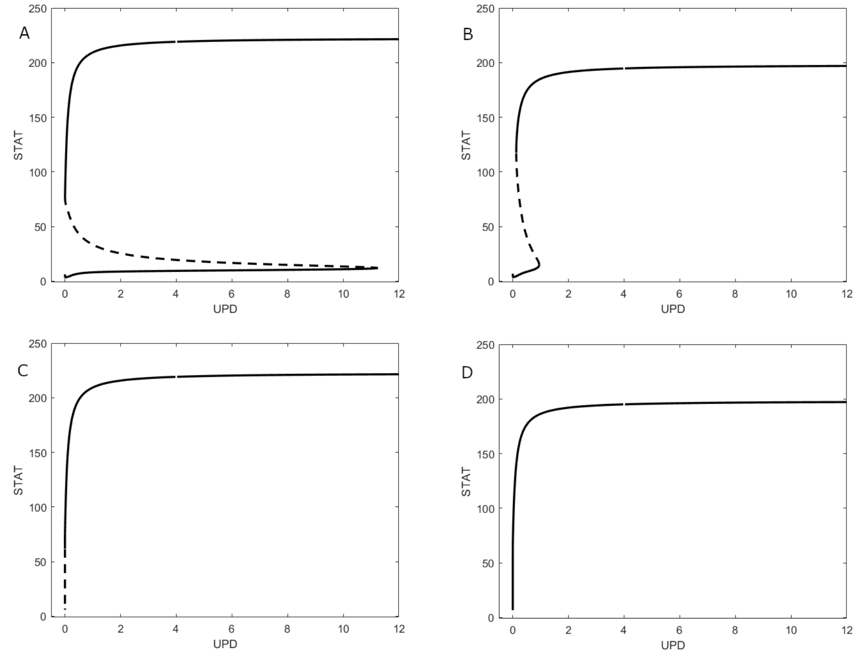

Figure S1. Bifurcation diagrams of STAT against UPD in three-variable model with (A) B^2 term and $k^f_{\beta R} = 100$ (B) B term and $k^f_{\beta R} = 100$ (C) B^2 term and $k^f_{\beta R} = 0$ and (D) B term and $k^f_{\beta R} = 0$.

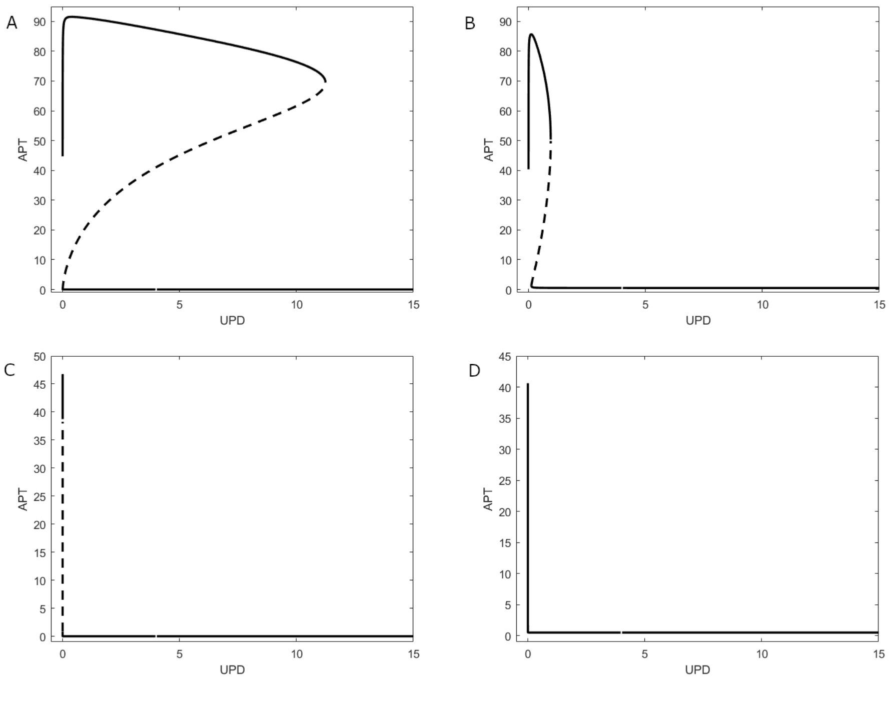

Figure S2. Bifurcation diagrams of APT against UPD in three-variable model with (A) B^2 term and $k^f_{\beta R} = 100$ (B) B term and $k^f_{\beta R} = 100$ (C) B^2 term and $k^f_{\beta R} = 0$ and (D) B term and $k^f_{\beta R} = 0$.

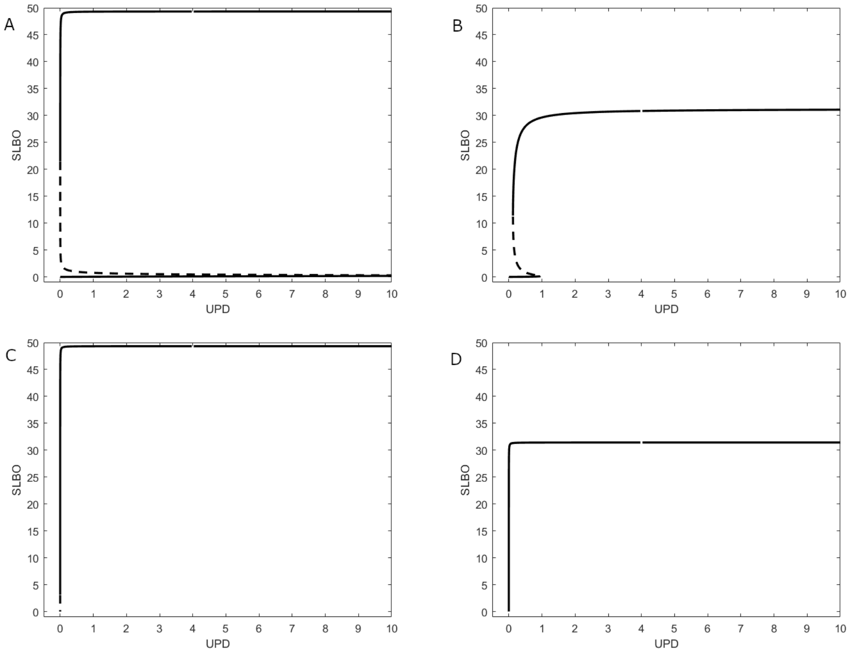

Figure S3. Bifurcation diagrams of SLBO against UPD in three-variable model with (A) B^2 term and $k^f_{\beta R} = 100$ (B) B term and $k^f_{\beta R} = 100$ (C) B^2 term and $k^f_{\beta R} = 0$ and (D) B term and $k^f_{\beta R} = 0$.

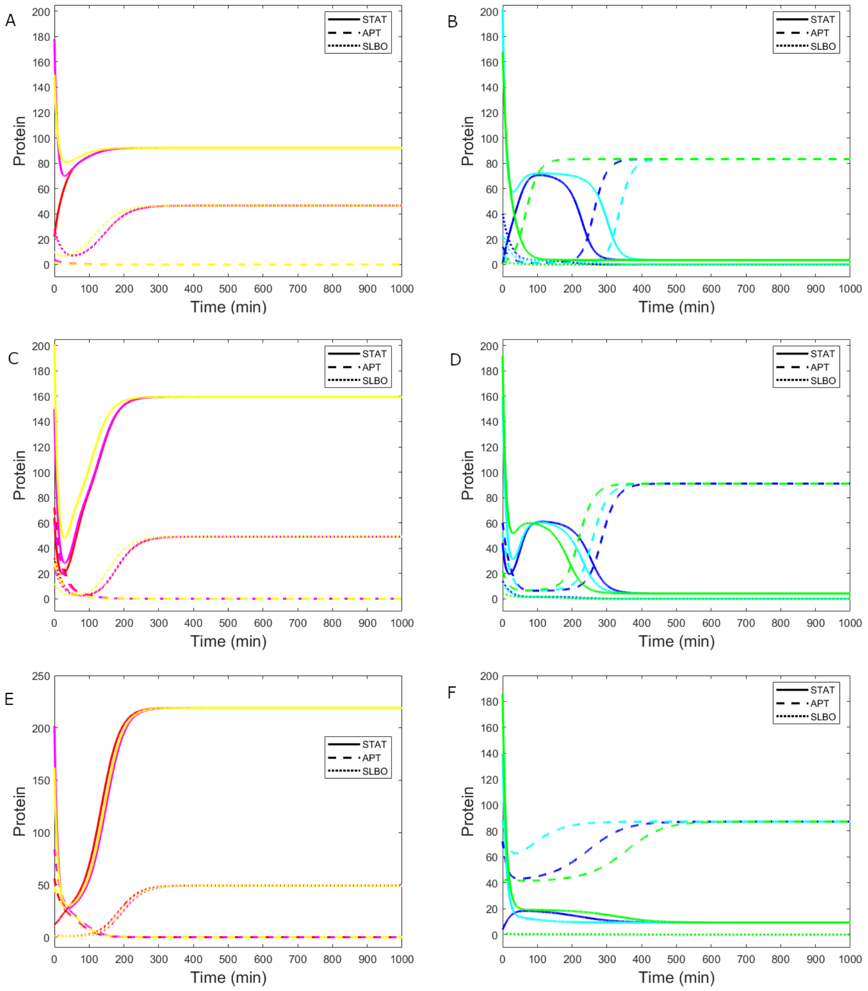

Figure S4. Time series of STAT, APT, and SLBO corresponding to trajectories in Figure 7. UPD=0.0133: (A) motile initial conditions (22,4,28), (178,4,28), and (150,1.4,10) and (B) stationary initial conditions (0,8,40), (202,4,27), and (168,0,0.5). UPD=0.133: (C) motile initial conditions (64,72,32), (150,60,25), and (201,28,12) and (D) stationary initial conditions (44,56,22), (160,52,21), and (192,22,8). UPD=4: (E) motile initial conditions (12,56,1.5), (202,84,2), and (162,48,1) and (F) stationary initial conditions (140,88,1.5),(4,72,1.4), and (186,36,0.4).

Rotating views of Figures 7A-C.

Figure S5. Video of Figure 7A

Figure S6. Video of Figure 7B

Figure S7. Video of Figure 7C

Rotating view of Figure 10

Figure S8. Video of Figure 9
